# Allogenic hematopoietic stem cell transplantation in two siblings with adult metachromatic leukodystrophy and a systematic literature review

**DOI:** 10.1002/jmd2.12221

**Published:** 2021-05-06

**Authors:** Cecilie Videbæk, Jette Stokholm, Henrik Sengeløv, Lone U. Fjeldborg, Vibeke Andrée Larsen, Christian Krarup, Jørgen E. Nielsen, Sabine Grønborg

**Affiliations:** ^1^ Neurogenetics Clinic and Research Lab Danish Dementia Research Centre, Rigshospitalet, University of Copenhagen Copenhagen Denmark; ^2^ Department of Paediatrics and Adolescent Medicine, Centre for Inherited Metabolic Disease Rigshospitalet, University Hospital Copenhagen Copenhagen Denmark; ^3^ Department of Clinical Genetics Rigshospitalet, University Hospital Copenhagen Copenhagen Denmark; ^4^ Bone Marrow Transplant Unit Copenhagen, Department of Hematology University of Copenhagen Copenhagen Denmark; ^5^ Hjerneskadecenter Virum Den Sociale Virksomhed Denmark; ^6^ Department of Radiology Rigshospitalet, University of Copenhagen Copenhagen Denmark; ^7^ Department of Clinical Neurophysiology Rigshospitalet, University of Copenhagen Copenhagen Denmark

**Keywords:** adult metachromatic leukodystrophy, hematopoietic stem cell transplantation, MRI‐MLD score

## Abstract

Two siblings were diagnosed with adult metachromatic leukodystrophy (MLD) and treated with hematopoietic stem cell transplantation (HSCT). While the older sibling was symptomatic at the time of diagnosis, her younger brother was diagnosed and transplanted at the presymptomatic state. We describe patients' clinical, biochemical, and genetic features, as well as neuropsychological and neurophysiological test results, and brain magnetic resonance imaging from pretransplantation and posttransplantation assessments. Both patients converted to complete donor chimerism and arylsulfatase A levels normalized 3 months posttransplantation. Twelve months posttransplantation, neurological and neuropsychological assessment for both patients showed stabilization, and they remained stable for the 38 months long observation period. To assess the effect of HSCT used as treatment for the rare, adult MLD subtype on survival and stabilization, we performed a systematic literature review and included 7 studies with a total of 26 cases. Of these 26 cases, 6 patients died of HSCT‐related complications and 2 patients had graft rejection. Of the remaining 18 patients, 2 patients improved after HSCT, 13 patients stabilized, and 3 patients progressed, suggesting that HSCT potentially benefits adult MLD patients. Larger studies focusing on this subtype are needed and recommendations on criteria for HSCT in adult MLD need to be evolved.

## 1 INTRODUCTION

Metachromatic leukodystrophy (MLD) is an autosomal recessive inherited lysosomal disease. It is caused by pathogenic variants in the *ARSA* gene encoding the enzyme arylsulfatase A, leading to accumulation of sulfatides and demyelination of the central and peripheral nervous system.^1^ MLD is characterized by progressive spasticity, ataxia, dementia, and early death, and in addition to these symptoms gall bladder mucosa can be affected.^2^

MLD is divided into three subtypes; the pediatric subtypes having onset before 30 months of age (late‐infantile) and before 16 years (juvenile), respectively, and the adult subtype with debut after 16. Generally, the adult form is the slowest progressing MLD subtype and onset is often with cognitive and psychiatric symptoms, followed by motor symptoms. With some geographical variation,^3^ adult MLD is present in approximately 20% of MLD cases, while late‐infantile is the most common subtype (40%‐50%).^4^

Hematopoietic stem cell transplantation (HSCT) was introduced as a possible treatment for MLD in the 1980s.^5^ It has been shown to stabilize disease progression in some MLD patients, but systematic outcome data are still missing.^6^ There is also varying success between the different MLD types with the infantile type not amenable for HSCT treatment^7^ but with better results for the juvenile form when transplantation is performed before symptom onset.^6^, ^8^, ^9^ Studies focusing on adult MLD are lacking and due to limited numbers, these patients often represent a relatively small part of larger cohorts, making it harder to draw conclusions about this specific subtype of MLD.^8^

## 2 CASE PRESENTATION

**FIGURE 1:**
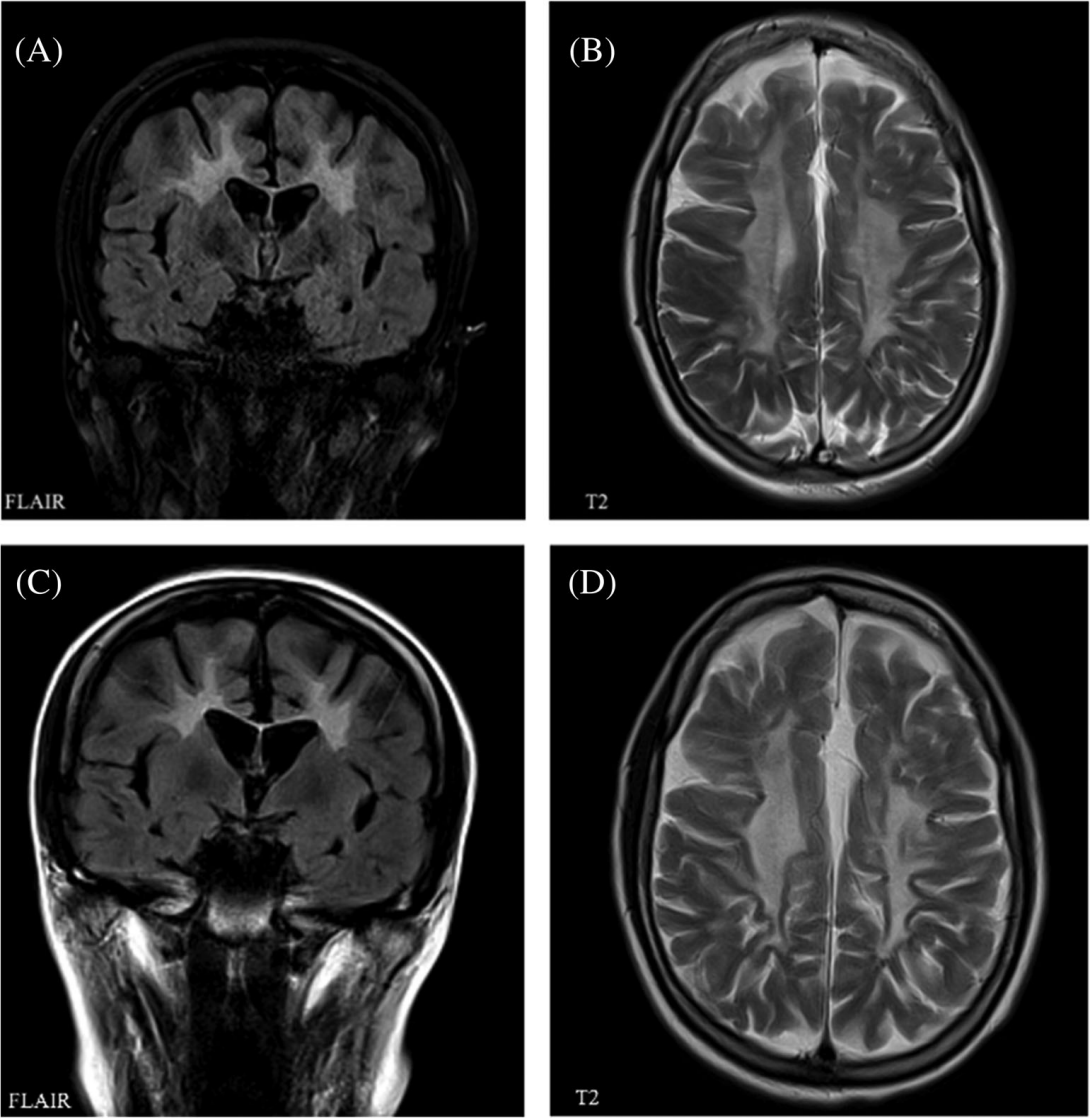
Brain MRI of patient A before and 36 months after transplantation. A (coronal section, FLAIR) and B (axial section, T2 weighting) show MRI performed 4 months before transplantation (age 20 years and 11 months), both showing widespread, symmetrical, and confluent supratentorial white matter changes typical of MLD and a MLD‐MRI score of 21/34. The typical tigroid pattern can be seen in B. C (coronal section, FLAIR) and D (axial section T2 weighting) show MRI performed 36 months posttransplantation (age 22 years and 9 months) with unchanged extensive white matter pathology, MLD‐MRI score unchanged of 21/34

*Patient A* is a 23‐year‐old woman with a history of presumed eating disorder as well as obsessive compulsive disorder (OCD)‐like symptoms from the age of 14 years. She had otherwise been healthy throughout childhood and had reached normal developmental milestones and unproblematic schooling until the age of 18 years. At this point progressive behavioral changes, mental slowing, difficulties concentrating, loss of initiative and motivation led to her obtaining the lowest possible grades in her A‐levels. During the same period, she experienced progressive difficulty in walking with staggering gait as well as pronounced difficulties walking stairs. Neurological examination at age 20 years and 5 months revealed reduced emotional control and increased latency, spastic‐ataxic, broad based gait (gross motor function classification in MLD [GMFC‐MLD] one),^10^ increased tone in upper and lower extremities with symmetrical hyperreflexia, and bilateral extensor plantar responses. Initial brain magnetic resonance imaging (MRI) showed diffuse and pronounced pathology of the white matter corresponding to an MRI‐MLD score of 21/34^11^ (Figure 1, Table S1). Neuropsychological evaluation showed a general cognitive impairment dominated by fluctuating attention, executive dysfunctions, and significant memory impairment. Full scale IQ was 70.^12^ The electrophysiological examination revealed a demyelinating polyneuropathy with slight axonal loss and somatosensory evoked potentials as well as motor‐evoked potentials showed mild central involvement (Tables S1 and S3). MLD diagnosis was confirmed by severely reduced arylsulfatase A activity (0,7 nmol/h/mg) (ref. 3.5–15 nmol/h/mg) and identification of homozygosity for the previously described pathogenic c.1277C>T; p.(P426L) variant in the *ARSA* gene. After careful consideration HSCT was performed 10 months after diagnosis and approximately 30 months after symptom onset (patient age 21 years, 3 months).

The patient received allogenic bone marrow transplantation from a 10/10 allele human leukocyte antigen (HLA)‐matched unrelated donor. Condition was myeloablative and consisted of busulfan 12.8 mg/kg, cyclophosphamide 120 mg/kg, and thymoglobulin 7.5 mg/kg. Rejection and graft‐versus‐host prophylaxis were applied with short‐course methotrexate and cyclosporine. Neutrophil engraftment was achieved by day 25. The posttransplantation course was uneventful. Cyclosporine was tapered to halt 1 year and 3 months after transplant. Donor chimerism 1 year after transplantation was >99% for the myeloid and >90% for the lymphoid lineage.

Arylsulfatase A levels measured 1.5 months after HSCT showed normalized levels (11.6 nmol/h/mg protein; ref. 3.5–15 nmol/h/mg). From 2 to 7 months post‐HSCT, the patient's motor function deteriorated slightly, probably due to increased spasticity (GMFC‐MLD) scale score 2).^10^ The patient received intensive neurorehabilitation from 7 to 17 months post‐HSCT and continued regular physiotherapy and activity of daily living training (37 months post‐HSCT). She regained ability for independent walking for short distances (10 m equivalent to GMFC‐MLD scale score 1).

Follow‐up brain MRI performed after 7, 14, 19, and 36 months did not indicate further disease progression (Figure 1). Nerve conducting velocity (NCV) performed 24 months after HSCT showed a very slight decline. Neuropsychological reevaluation after 8, 15, and 36 months showed stable scores on subtests from Wechsler Adult Intelligence Scale (WAIS‐IV) over time with a General Ability Index of 68 and 67 (Table S1). Due to severe executive difficulties, the patient's functional level was severely impaired. During the rehabilitation program, she improved regarding memory, attention, and the ability to maintain a conversation, while her initiative continued to be impaired, but has improved. Thirty‐six months after HSCT, the patient is in a stable condition and is preparing to move to a residence for young people with acquired brain damage.

*Patient B* is the younger brother of patient A. Predictive molecular genetic testing at age 17 revealed homozygosity for the c.1277C>T; p.P426L variant in the *ARSA* gene and arylsulfatase A activity was reduced (0.7 nmol/h/mg) (ref. 3.5–15 nmol/hour/mg). The patient was performing well in his second year of high school and did not have motor complaints or psychiatric symptoms.

**FIGURE 2:**
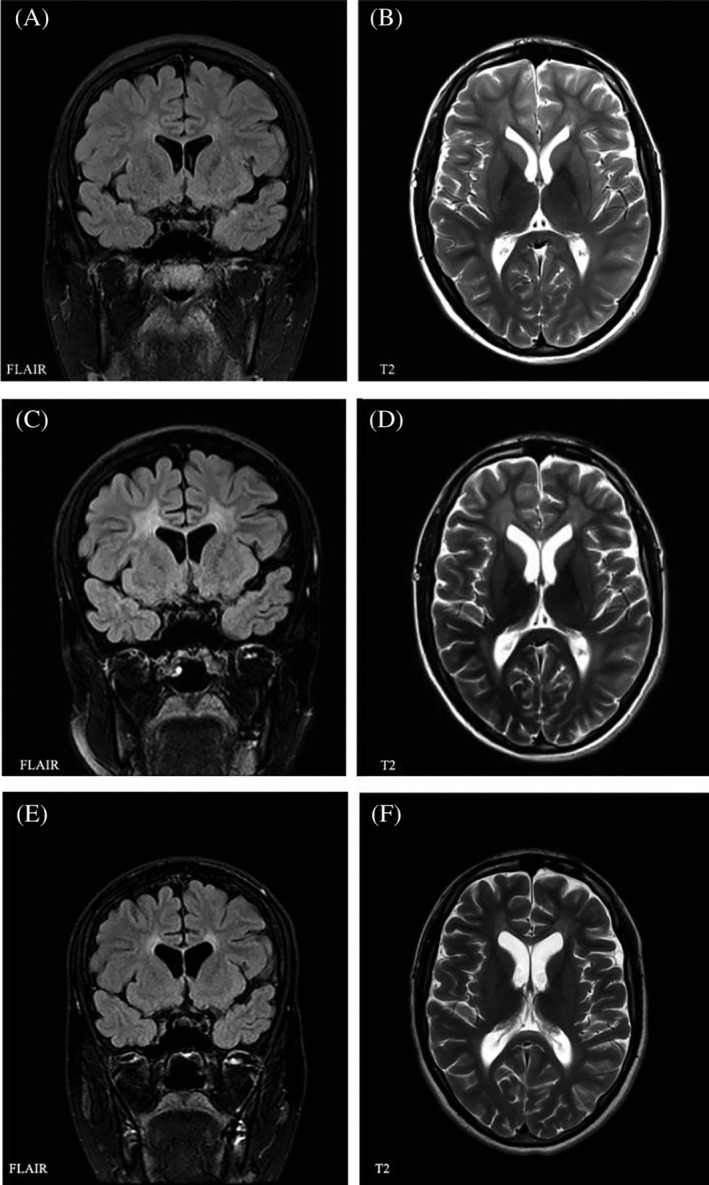
Brain MRI patient B: Brain MRI of patient B before HSCT and 9 and 37 months after. A (Coronal section, FLAIR) and B (Axial section, T2 weighting) were performed one month before transplantation (age 18 years) showing mild white matter pathology in the frontal and parieto‐occipital region as well as the periventricular and central region corresponding to an MLD‐MRI score of 5/34. C (Coronal section, FLAIR) and D (Axial section, T2 weighting) were performed 9 months after transplantation (age 18 years and 10 months) showing symmetrical, periventricular white matter changes in the cerebral hemispheres as well as a slight involvement of U‐fibres in few gyri. The findings correspond to an MLD‐MRI score of 13/34. E (Coronal section, FLAIR) and F (Axial section, T2 weighting) were performed 37 months after transplantation (age 19 years, 9 months) showing a decreased distribution and intensity of white matter pathology, no longer with involvement of U‐fibres. The findings correspond to an MLD‐MRI score of 11/34

Neurological examination revealed absent Achilles tendon reflexes but was otherwise normal. On brain MRI faint periventricular white matter hyperintensities was noted (MLD‐MRI score of 5/34; Figure 2). Nerve conduction studies showed signs of a demyelinating polyneuropathy, with only marginal signs of axonal loss. Neuropsychological evaluation revealed an IQ of 97.^12^ Performance in tests of processing speed (Trail Making Tests A and B and verbal fluency) as well as tests of learning and recall (Rey Auditory Verbal Learning Test and Rey Figure Test) was likewise normal (Table S2). The patient's MLD disease status was considered presymptomatic, and HSCT was performed 7 months after diagnosis (age 18 years, 1 month).

The patient received allogenic bone marrow transplantation from a 10/10 allele HLA‐matched unrelated donor, following the same protocol as his sister. Neutrophil engraftment was achieved on day 23. He encountered an episode of self‐limiting hemorrhagic cystitis, otherwise the posttransplantation course was uneventful. Cyclosporine was tapered to halt 1 year and 5 months after transplantation. Donor chimerism 1 year after transplantation was >99% for the myeloid and >70% to 90% for the lymphoid cell populations. Due to development of exocrine pancreatic insufficiency, oral enzyme supplementation was provided.

One month after HSCT arylsulfatase A activity was normalized (14.5 nm/h/mg protein [ref. 3.5–15 nmol/h/mg protein]). Brain MRI 9 months after HSCT showed an increase in white matter pathology (MRI‐MLD score of 13/34; Figure 2),^11^ which had decreased in intensity and distribution in all regions at 14, 21, and 37 months after HSCT (MRI‐MLD score 11/34; Figure 2).^11^ Magnetic resonance cholangiopancreatography 11 months after HSCT, performed because of an inconclusive ultrasound result, showed slight thickening of the gallbladder wall. Neurological examination 5 and 12 months after transplantation was normal including normalized Achilles tendon reflexes. Nerve conduction velocities were unchanged as was neuropsychological evaluation 8 and 22 months post‐HCST (Table S4). Thirteen months after transplantation, the patient returned to high school performing with average results. During the observation period of 38 months post‐HSCT, no clinical signs of disease progression have been noted.

## 3 LITERATURE REVIEW

### 3.1 Methods

We searched PubMed through 1 December 2019. We used the medical subject heading terms “Metachromatic,” “Leukodystrophy,” and “MLD” in combination with “Adult,” “Late onset,” and “HSCT,” “Transplantation,” “Bone marrow transplantation.” We only included studies that reported a definite genetic or enzymatically confirmed diagnosis of MLD with onset at >16 years, included HSCT treatment, provided data on each individual case, and were published in English. We included one study by Van Rappard et al where information about exact age at onset and age at HSCT were not stated.^6^ Altogether we included 7 studies resulting in a total of 26 cases.^5^, ^6^, ^8^, ^13^, ^14^, ^15^, ^16^

## 4 RESULTS

Symptom onset ranged from 16 to 41 years of age (median: 19 years; age at symptom onset was missing for 6 patients^6^). Diagnosis was supported by reports of pathogenic *ARSA* variants and/or reduced arylsulfatase A enzyme activity in 20 cases. For 6/26 patients^6^ arylsulfatase A activity in leukocytes was monitored but not reported.

In the seven studies, evaluation of patient outcome after HSCT was based on brain MRI,^5^, ^6^, ^8^, ^13^, ^14^, ^15^, ^16^ neurophysiology,^13^, ^15^, ^16^ neuropsychology,^6^, ^13^, ^14^, ^15^, ^16^ and/or clinical findings. Patients' motor function was not described in a formalized manner. In order to compare patient characteristics, the patients were divided into three groups, according to their outcome after HSCT as assessed by the original authors of the studies (progressed, stabilized, improved) (Table 1). It was necessary to group patients according to the original authors' evaluation, because defining outcome criteria for evaluation of MLD patient course after HSCT are yet to be agreed on. Also, reported outcome measures varied between the seven studies and often objective quantitative parameters were lacking. As an example, MLD‐MRI score was presented in two studies only^5^, ^6^ (see details in Table 1), and only one study provided a score both before and after transplatation.^6^ In the remaining five studies, MRI was either described or simply labeled as improved/stabilized/progressed. Only two papers described their patients (n = 2) as having improved.^15^, ^16^ This was in regard to cognitive function^15^, ^16^ and neurophysiological testing.^15^ Stabilization of patients is documented by the clinical case description in most of the cases (missing in detail in Boucher et al^8^) while MRI findings (12/13 cases^6^, ^8^, ^13^, ^16^), testing of cognitive functions (7/13^6^, ^13^, ^16^), and neurophysiological testing (8/13^8^, ^13^, ^16^) are used as documentation as well. Stabilization usually occurred within a year. Progression of the patients is documented similarly with MRI evaluation for 3/3,^5^, ^13^, ^14^ testing of cognitive functions for 2/3,^13^, ^14^ and neurophysiology for 1/3^13^ patients. Overall, 67% of the cases included in this review were described with a positive outcome after HSCT with either stabilization or improvement during the mean follow‐up period of 72 months. The remaining 33% either progressed or died from complications related to transplantation. In the cases where debut symptoms were available, all cases were reported to have had debut with cognitive or behavioral symptoms, with the exception of two cases who debuted with motor symptoms.^16^

**TABLE 1:** Characteristics of included cases grouped according to their outcome

	All patients (n = 26)	Patients with improvement (n = 2)	Patients with stabilization (n = 13)	Patients with progression (n = 3)	Patients who died^a^ (n = 6)	Patients who rejected^b^ graft (n = 2)
Age at symptom onset in months (median)	192‐492 (228)	216‐288 (252)	192–492 (294)	216‐300 (216)	192‐476 (342,5)	300
Time from symptom onset to HSCT in months (median)	11‐120 (48)	13–48 (35)	36‐130 (62)	18‐108 (72)	0‐109 (24)	48
Patients who were presymptomatic at transplant	0	0	0	0	1	0
Donor type	Unrelated: 12 Umbilical cord: 5 Related: 3 6 missing data	Unrelated: 1 Umbilical cord: 0 Related: 1	Unrelated: 5 Umbilical cord: 2 Related: 1 5 missing data	Unrelated: 2 Umbilical cord: 0 Related: 1	Unrelated: 2 Umbilical cord: 3 Related: 0 1 missing data	Unrelated: 2 Umbilical cord: 0 Related: 0
Observation time (months)	1‐216 (72)	48	13‐144 (80.5)	18‐132 (108)	1–77 (2.5)	
MRI		1/2 remained stable^16^ 1/2 was missing follow‐up data^15^	7/13^6^, ^8^, ^13^, ^16^ remained stable 2/13 improved^6^, ^8^ 3/13 had very slight deteriorations^6^ Missing data: 1^8^ Loes score pre‐HSCT and post‐HSCT available for 5 patients, changes varied from −1 to +4^6^	2/3 showed progression^13^, ^14^ Missing data: 1^5^		
Cognitive function		1/2 improved (full‐scale IQ from 52 to 73 and verbal IQ from 61 to 89)^15^ 1/2 remained stable in testing, but clinical assessment slightly improved^16^	3/13 remained stable^6^, ^16^ 4/13 declined slightly^6^, ^13^ Missing data: 6^8^	2/3 declined post‐HSCT^13^, ^14^ Missing data: 1^5^		
Neurophysiology		2/2 improved^15^, ^16^	3/13 remained stable^13^, ^16^ Missing data: 10	1/3 declined in sensory NCS^13^ Missing data: 2^5^, ^14^		
References		15, 16	6, 8, 13, 16	5, 13, 14	6, 8, 13	13

Abbreviations: HSCT, hematopoietic stem cell transplantation; MRI, magnetic resonance imaging; NCS, nerve conduction study.

^a^ 6/26 patients died after HSCT, for 4/6 cause of death was stated and was related to transplantation complications, 1/6 died due to disease progression and for 1/6 cause of death was unknown.

^b^ 2/26 patients had graft rejection and were not re‐transplanted due to poor clinical condition.

In the study by Van Rappard et al^6^ 6/7 patients stabilized after HSCT and one patient progressed. Saute et al^5^ presented one case with adult MLD who continued to progress after transplantation. Solders et al^15^ reported improved peripheral nerve conduction, electroencephalogram and verbal IQ after HSCT in one patient. Solders et al^16^ reported another case with improved cognitive function as well as two patients who stabilized after HSCT performed at 108 and 36 months, respectively, after symptom onset. Likewise, Boucher et al^8^ reported five patients who all stabilized despite having symptoms at the time of transplantation. In this study four additional patients died, three of transplantation‐related causes and in the last case, the cause was unknown. In a case reported by Smith et al,^14^ a patient progressed after HSCT 6 years after symptom onset. De Hosson et al^13^ reported five cases of adult MLD. One patient had graft rejection and primary graft failure occurred in another patient. One patient died of graft vs host disease only 35 days after transplant. Of the remaining two patients, one progressed and one stabilized.

## 5 DISCUSSION

Division of MLD subtypes is based on age at symptom onset, but the first symptoms can be difficult to assess, especially in the slower progressing forms and in regard to psychiatric and cognitive symptoms. Patient A was suspected of early psychiatric symptoms at the age of 14 (anorexia and OCD) but had no definitive symptoms of MLD until 18 years of age (pronounced decline in school performance). The symptoms could, however, potentially have been early signs of MLD, in which case patient A would have been categorized as late‐onset juvenile MLD patient. Her younger brother (patient B) was asymptomatic at the time of HSCT, aged 18 years and 1 month, suggesting possible intrafamilial variability of this late onset disease course. Even for *ARSA* gene variants with a clear correlation between subtype and genotype^3^ variability in age of onset has been observed in both unrelated cases and in siblings.^17^ Intrafamilial variability for age of onset, but not for initial symptoms and disease progression, has been confirmed mostly for the juvenile and adult subtypes, with age of onset differing up to 6 and 9 years in juvenile and adult MLD sibling pairs, respectively.^18^

In both our patients, we have not seen progression after HSCT neither clinically nor on brain MRI after 14 months post‐HSCT (Figures 1 and 2). Likewise, neuropsychological test results (Table S1) were stable, and we considered significant retest effect as unlikely. Generally, disease stabilization is expected within 6 to 24 months after HSCT.^16^, ^19^, ^20^ On brain MRI, patient B progressed from MRI‐MLD score^11^ 5 to 13/34 in the posttransplantation period. MRI‐MLD score had improved again (11/34) already 5 months later (Figure 2). Previous reports have stated that faster progression of MLD can be seen during the transplantation period,^13^, ^16^ including temporary deterioration followed by MRI improvement in MLD cases that underwent HSCT in early stages of disease.^21^ Initial deterioration on MRI has been thought to be due to further disease progression and possibly effects of HSCT itself before stabilization occurs.^21^ We cannot exclude that the pronounced, clinically silent deterioration of MLD‐MRI score in the first 9 months after HSCT was aggravated by HSCT related toxicity. Stabilization and improvement of MRI 14 months after HSCT is in line with previous observations.^9^, ^19^, ^21^ Interestingly, it appears that measurement of demyelination load and ^1^H‐MRS abnormalities could be more sensitive parameters, which in some cases show further improvement even after MLD‐MRI score stabilized.^19^, ^21^ Nerve conducting studies in both patients indicated a sensory and motor polyneuropathy with little or no signs of axonal loss (Tables S3 and S4). Patient B remained stable in nerve conducting studies, but patient A deteriorated slightly after transplantation (24 months post‐HSCT) in regard to NCV. The deterioration was very mild and without clinical symptoms. This is in line with recent description, in which NCV did not change significantly in 12 juvenile MLD patients in up to 5 years after HSCT, regardless of preexisting neuropathy.^22^ It is expected that gene therapy approach might be more effective for peripheral neuropathy treatment due to achievement of higher enzyme levels,^23^ as might be the case with intravenous enzyme replacement therapy.^24^

Generally, early and presymptomatic HSCT for MLD treatment is expected to provide better treatment outcome.^9^ Since these siblings were transplanted at different MLD disease stages, they provide an interesting perspective for HSCT indication in adult MLD patients with slower disease progression. Longer observation time is needed to make final conclusions, but during our observation time of 38 months, HSCT has halted the progression of MLD in both siblings, transplanted at symptomatic and presymptomatic disease stages. It has recently been observed that patients who remain stable within the first 12 to 24 months after HSCT are more likely to have a sustained stable course.^22^ This supports possible sustained stable disease course in our two cases, in spite of limited observation time so far.

The following criteria for HSCT were suggested for juvenile MLD: (1) clinically presymptomatic or early symptomatic (GMFC‐MLD score of 0 or 1 and IQ ≥85); (2) brain MRI severity score^11^ less than 17 (with a temporal or parietooccipital white matter sub score ≤4) and no involvement of U‐fibers; and (3) Onset age older than 4 years.^9^ Only one patient included in our review was transplanted at a presymptomatic state,^8^ but died shortly after HSCT due to complications. Despite symptomatic state, 15/26 patients had positive outcome from HSCT suggesting that presymptomatic state might not be mandatory to obtain positive effect in adult‐onset MLD patients. Median time from initial symptoms to transplantation was 48 months, while median time from diagnosis to transplantation was 12 months. This emphasizes that long diagnostic delay still is a major challenge. Initially slowly progressive psychiatric symptoms lead to misdiagnosis in a substantial number of patients.^25^ Accordingly, more attention is needed on early diagnosis and general awareness of adult MLD needs to be increased, so that HSCT can be discussed in the early phase.

In our review of the literature, 6/26 (23%) cases died during or after HSCT. Four of these patients were from the same cohort, described by Boucher et al,^8^ and in 3, the cause was transplantation related (unknown cause in fourth patient). The authors point out that a significant number of the patients were treated in an era when HSCT was associated with higher mortality, which might be an important contributing factor to overall mortality in our review.^8^ Twenty‐three percent mortality, however, is similar to the mortality rate for HSCT in juvenile MLD reported in one other study (79% 5‐year survival).^9^ In this study, 67% of the deaths were due to transplantation complications and the remaining were due to rapid progression. Early rapid disease progression that has been observed in a subset of patients after HSCT could be one important factor for mortality in HSCT for MLD.^22^

In the proposed transplantation criteria for juvenile MLD IQ cutoff is 85.^9^ In the adult MLD studies reviewed, an IQ criterion was used only in one study (IQ 70).^6^ In this study, 6/7 patients (83%) stabilized after HSCT (mean follow‐up 4.7 years). Two patients had an IQ lower than 85: one stabilized with an IQ of 72, while one had an IQ of 61, was included despite cutoff, and progressed. One other case, in our review had an IQ lower than 85^15^ and this patient improved after HSCT.^15^ Furthermore, an IQ of 85 is within the normal IQ range,^12^ and a transplantation criterion of 85 might exclude patients that do not have cognitive decline. MLD‐MRI scores for the patients reviewed were all under 17, except 1 patient (MLD‐MRI score 20, progressive course post‐HSCT^5^). Patient A in our report had a full‐scale IQ of 70 and MLD‐MRI score of 21 as well as involvement of U‐fibers but remained stable in clinical, MRI, and neuropsychological evaluations after HSCT. Although IQ and MLD‐MRI score are not sufficient for evaluating a patient's eligibility for transplantation on their own, this might suggest that HSCT in adult MLD patients can be considered at slightly more progressed disease states compared to juvenile MLD. Recently, motor symptoms at disease onset in juvenile and adult MLD cases were identified to predict rapid disease progression.^26^ The type of neurological symptoms at disease onset (motor vs cognitive) could thus be included into an algorithm to evaluate HSCT amenability as well. Noteworthy, we observed that the median age of symptom onset was later in the patient group that stabilized (2 patients) and in the group that improved (13 patients) (6.5 and 3 years later, respectively; Table 1) as compared to the group with unfavorable outcome after HSCT (9 patients). These results could be influenced by the fact that later disease onset generally indicates slower disease progression, possibly indicating a wider therapeutic window also within the adult MLD patient group.

Together with lack of natural history data and pronounced phenotypic variability of adult MLD, one major challenge drawing conclusions across studies in this review was that reported outcome measures varied widely. In order to develop specific HSCT criteria for adult MLD, it is crucial to determine patients' MLD disease status, understand progression rate for individual cases and to achieve consensus for outcome reporting. Objective and quantifiable data concerning age at symptom onset, genotype, age at HSCT, MRI‐MLD score, clinical status including GMFC‐MLD score for motor function, neuropsychological and electrophysiological data and HSCT specific information need to be collected and collection of additional systematic data, for example, regarding quality of life, should be considered. Larger studies including the adult subtype are needed and recommendations on criteria for HSCT should be evolved in an international collaborative effort. Generally, slower progression seems to make adult MLD amenable for HSCT treatment, and in combination with initiatives increasing disease awareness and early diagnosis, HSCT may have a significant impact on disease burden in this patient group in the future.

## CONFLICT OF INTEREST

The authors declare no potential conflict of interest.

## AUTHOR CONTRIBUTIONS

*Conception and design*: Cecilie Videbæk, Jørgen E. Nielsen, Sabine Grønborg. *Analysis and interpretation of data, drafting, and revising of manuscript*: Cecilie Videbæk, Jette Stokholm, Henrik Sengeløv, Lone U. Fjeldborg, Vibeke Andrée Larsen, Christian Krarup, Jørgen E. Nielsen, Sabine Grønborg. *Guarantor*: Sabine Grønborg.

## ETHICS STATEMENT

Our institution did not require ethics approval of this study.

## INFORMED CONSENT

The patients have given informed consent regarding publication of case reports and illustrations.

## ANIMAL RIGHTS

This article does not contain any studies with human or animal subjects performed by the any of the authors.

## Supporting information


**Table S1:** Paraclinical assessments of patient A before and after hematopoietic stem cell transplantation
**Table S2**. Paraclinical assessments of patient B before and after hematopoietic stem cell transplantation
**Table S3**: Electrophysiology for patient A before and after hematopoietic stem cell transplantation
**Table S4**: Electrophysiology for patient B before and after hematopoietic stem cell transplantation
