# Supplementary material for: Allogenic hematopoietic stem cell transplantation in two siblings with adult metachromatic leukodystrophy and a systematic literature review
Source: JIMD Rep. 2021 May 6;60(1):96–104. doi: 10.1002/jmd2.12221 (PMC8260480; doi:10.1002/jmd2.12221)

Supplementary Material

Table S1. Paraclinical assessments of patient A before and after hematopoietic stem cell transplantation

|  | Pre-transplantation | Post-transplantation |
| --- | --- | --- |
| MRI | **MRI 9, 4 and 1 months pre-transplantation**  Eichler score unchanged: 21/34.  Sub-scores: frontal WM: 2/2/1; parieto-occipital WM: 2/2/1; temporal WM 2/1/0; Corpus callosum: 2/2, projection fibers: 1/0/1; cerebral atrophy 1; thalamus and basalganglia: 0/0; Cerebellum 0/1 | **MRI seven, 14 and 19 months post-transplantation**  Eichler score unchanged: 21/34. Sub-scores unchanged |
| Electrophysiology  (See appendix for full data) | **9 months before transplantation**  Conclusions: severe sensory-motor polyneuropathy. Slow motor conduction velocity and increased F-wave latency consistent with demyelinating neuropathy. Considerable loss of sensory fibres. | **24 months post-transplantation**  Slight deterioration with further slowing of conduction velocity. |
| Arylsulfatase A activity  ref. 3,5-15 nmol/hour/mg protein | **9 months before transplantation:**  Lkc: 0,7 nmol/h/mg protein | **18 months post-transplantation:** 14,4 nmol/h/mg protein |
| Neuropsychology | **5 months before transplantation**  WAIS-4 index scores:  Verbal understanding: 75  Perceptual reasoning: 74  Working memory: 70  Processing speed: 68  Overall IQ: 70  GAI*: 72 | **15 months post-transplantation:**  WAIS-4 Index scores:  Verbal understanding: 77  Perceptual reasoning: 66  Working memory: 79  GAI*: 67 |

White matter = WM., Wechsler Adult Intelligence Scale, fourth edition = WAIS-4. * Due to increased impairment of fine motor skills after the transplantation, tests of processing speed could not be performed. General Ability Index (GAI) was therefore used instead of Full IQ

Table S2. Paraclinical assessments of patient B before and after hematopoietic stem cell transplantation

|  | Pre-transplantation | Post-transplantation |
| --- | --- | --- |
| MRI | **1 and 3 months pre-transplantation**  Eichler score: 5/34.  Sub-scores: frontal WM: 1/1/0; parieto-occipital WM: 1/1/0; temporal WM 0/0/0; Corpus callosum: 1/0, projection fibers: 0/0/0; cerebral atrophy 0; thalamus and basalganglia: 0/0; Cerebellum 0/0 | **9 months post-transplantation**  Eichler score: 13/34.  Sub-scores: frontal WM: 2/2/1; parieto-occipital WM: 2/2/0; temporal WM 0/0/0; Corpus callosum: 2/1, projection fibers: 0/0/0; cerebral atrophy 1; thalamus and basalganglia: 0/0; Cerebellum 0/0  **14 and 21 months post-transplantation**  Eichler score: 11/34.  Sub-scores: frontal WM: 2/1/0; parieto-occipital WM: 2/1/0; temporal WM 0/0/0; Corpus callosum: 2/2, projection fibers: 0/0/0; cerebral atrophy 1; thalamus and basalganglia: 0/0; Cerebellum 0/0 |
| Electrophysiology (See appendix for full data) | **2 months before transplantation**  Conclusion: Reduced sensory and motor conduction velocities with only marginal signs of axonal loss consistent with demyelinating sensory-motor neuropathy. | **25 months post-transplantation**  Conduction velocities and amplitudes were unchanged |
| Arylsulfatase A activity (ref. 3,5-15 nmol/hour/mg protein) | **7 months before transplantation:**  Arylsulfatase A, Lkc: 0,7 nmol/hour/mg protein | **20 months post-transplantation:**  Arylsulfatase A, Lkc: 14,5 nmol/hour/mg protein |
| Cognitive function | Normal cognitive functions, IQ: 97 | **8 and 22 months post-transplantation:**  No changes in cognitive functions |

White matter = WM.

Table S3: Electrophysiology for patient A before and after hematopoietic stem cell transplantation.


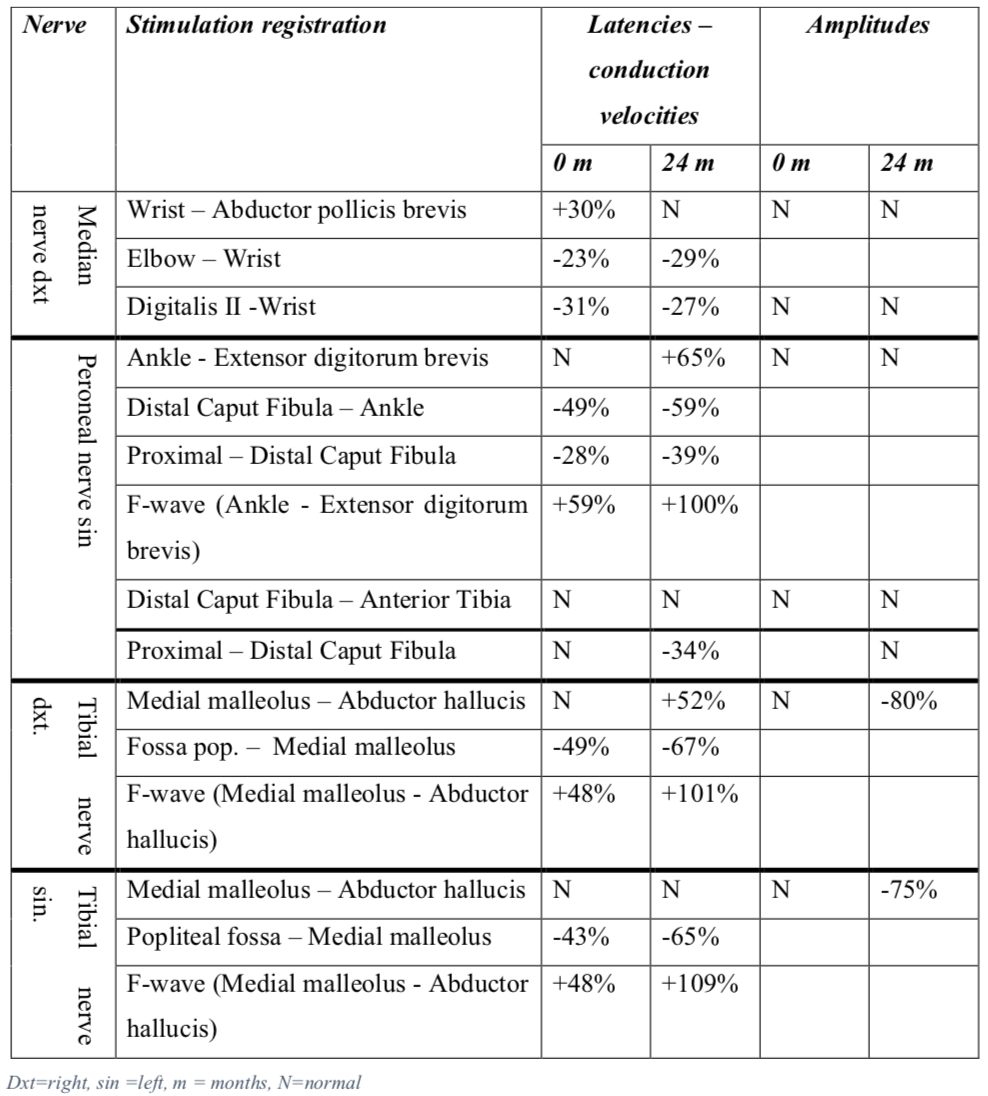


Table S4: Electrophysiology for patient B before and after hematopoietic stem cell transplantation.


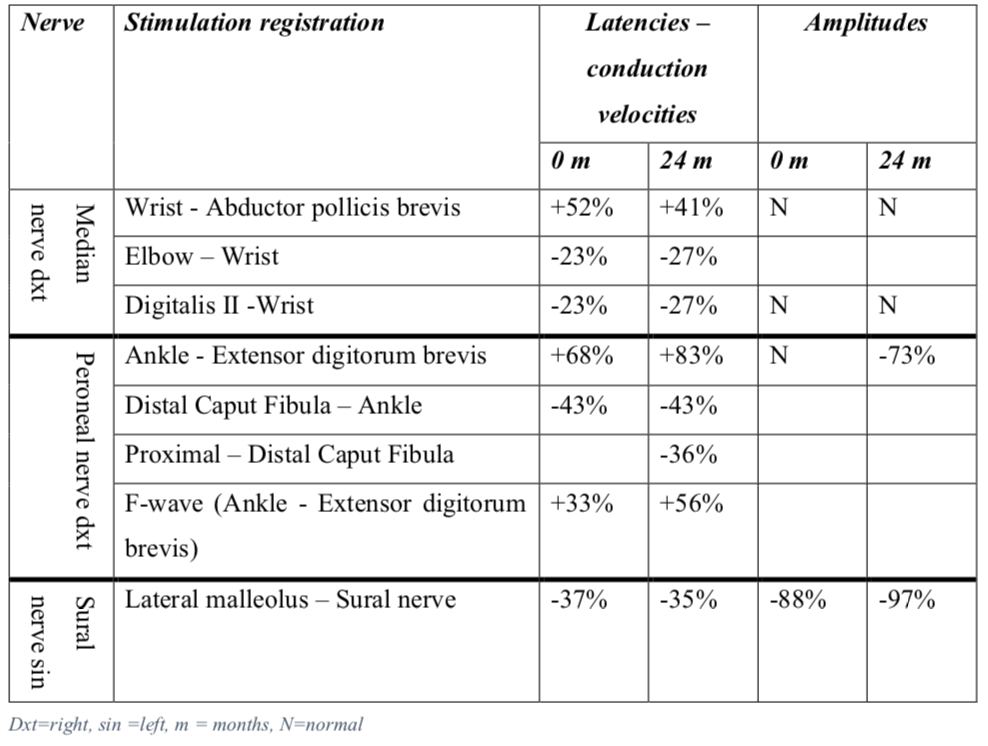

Supplement: Supplementary file 1 — Table S1: Paraclinical assessments of patient A before and after hematopoietic stem cell transplantation Table S2. Paraclinical assessments of patient B before and after hematopoietic stem cell transplantation Table S3: Electrophysiology for patient A before and after hematopoietic stem cell transplantation Table S4: Electrophysiology for patient B before and after hematopoietic stem cell transplantation [file JMD2-60-96-s001.docx]
